# Microbiome legacy influences necrosis formation in *Diplodia sapinea*-infected Scots pine shoots

**DOI:** 10.1186/s40793-026-00904-9

**Published:** 2026-05-09

**Authors:** Dániel G. Knapp, Alexandra Nagy, Elham Badalzadehe, Anna Molnár, Johan Kroon, Carmen Romeralo, Julio Javier Diez, Johanna Witzell

**Affiliations:** 1 Department of Forestry and Wood Technology, Linnaeus University, Växjö, Sweden; 2 Food and Wine Research Centre, Eszterházy Károly Catholic University, Eger, Hungary; 3 Skogforsk – The Forestry Research Institute, Ekebo, Sweden; 4 Institute of Forest Sciences (ICIFOR), INIA-CSIC, Madrid, Spain; 5 University of Valladolid, Valladolid, Spain

**Keywords:** Diplodia tip blight, Necrotic lesions, Fungal microbiome, *Sphaeropsis sapinea*, Third-generation sequencing

## Abstract

**Background:**

Fungal endophytes are important members of the holobiont of all plants, including that of Scots pine (*Pinus sylvestris*), potentially affecting host performance. One of the most important pathogens of Scots pine in Europe is *Diplodia sapinea*, which causes necrotic lesions and is becoming increasingly prevalent in northern regions. Although endophytes are known to affect plant performance, it remains unclear whether naturally established fungal communities in Scots pine shoots can modulate *D. sapinea*-induced necrosis. Using a field experiment, we tested the hypothesis that exclusion of airborne fungal inoculum shapes the endophytic community in shoots of pine seedlings, and that such alterations in this community influence the necrosis-inducing capacity of *D. sapinea*.

**Results:**

In the field site, airborne fungal inoculum was reduced in half of the saplings by covering shoots with mesh bags. Covered (bagged) and free (unbagged) shoots were transported to the laboratory and inoculated with *D. sapinea*. The morphology and physiological status of the shoots were monitored using a multispectral 3D scanner, and the necrotic lesion development was assessed. The propagule exclusion resulted in endophytic communities with slightly lower richness, while shoots showed no detectable morphological or physiological differences prior to inoculation. Shoots inoculated with *D. sapinea* developed clear necrotic lesions, which were significantly larger in covered shoots than in the free ones. Long-read Oxford Nanopore metabarcoding revealed that community shifts following inoculation were more pronounced in covered shoots. Community composition clearly separated necrotic and healthy tissues.

**Conclusions:**

Our findings suggest that the structure of the resident fungal endophytic community may influence the extent of necrotic lesions caused by *D. sapinea* in Scots pine shoots. A more established, diverse fungal community was associated with smaller lesion sizes, whereas shoots exposed to lower propagule pressure developed larger lesions following inoculation. These results highlight the functional role of fungal community assembly in shaping disease outcomes and suggest that endophyte-based approaches may provide new opportunities for improving disease resistance in forest tree species. The results also suggest that endophytic status may need to be considered when lesion size is used to evaluate resistance to pathogens in tree breeding programs.

**Supplementary Information:**

The online version contains supplementary material available at 10.1186/s40793-026-00904-9.

## Background

Land plants live in association with various asymptomatically present microorganisms within their tissues. This microbiome, together with the host, is referred to as the plant holobiont, in which the partners jointly face challenges from biotic and abiotic stresses, as well as defense mechanisms [1–3]. Key members of the microbiome in healthy plants are fungi residing in inner tissues, generally referred to as fungal endophyte*s*. These fungi colonize plant tissues without causing observable damage during at least part of their life cycle [4, 5]. The endophytic communities consist of various taxa with different lifestyles, such as latent saprotrophs or latent pathogens, but accumulating evidence indicates that they can also contribute directly or indirectly to plant defense against microbial pathogens [6, 7]. Moreover, several fungal taxa are known to shift from an endophytic to a pathogenic lifestyle when host conditions change, highlighting the context-dependent nature of these associations [8].

The combined genetic repertoire of a plant’s associated microbial community is larger than that of the plant itself [9]. The functional importance of this dynamic “second genome” may be particularly important in perennial, long-lived plants like forest trees. In Europe, Scots pine (*Pinus sylvestris*) is an environmentally, economically and culturally important forest tree species, covering 28 million hectares and representing about 20% of the European Union’s commercial forest area [10, 11]. Across its wide distribution area, Scots pine is increasingly threatened by fungal pathogens, causing destructive diseases such as Pine Blister Rust (*Cronartium* spp.) [12–14] and Diplodia Tip Blight, DTB, caused by *Diplodia sapinea* (Fr.) Fuckel (syn. *Diplodia pinea* (Desm.) Kickx. and *Sphaeropsis sapinea* (Fr.: Fr.) Dyko & Sutton) [15, 16]. *Diplodia sapinea* is repeatedly reported as an opportunistic necrotrophic pathogen on conifers, especially *Pinus* species [16–20]. Although it is globally distributed [16], reports of DTB have increased in recent years in the northern hemisphere, particularly in Europe [16, 19–22]. The pathogenicity of *D. sapinea*, which may also act as a common endophyte [23], tends to increase when the host tree faces abiotic stressors such as drought or mechanical damage [20, 24].

More detailed knowledge of the processes shaping fungal endophyte communities in pine shoots may contribute to the development of microbiome-based strategies to support pine health in a changing risk landscape. This potential is illustrated by successful bio-based controls already used in forestry [25]. Although progress has been made, our knowledge of the establishment and diversity of fungal endophytic communities in Scots pine, particularly those inhabiting inner stem and branch tissues, remains limited. However, current evidence suggests that the majority of the endophytes originate from airborne fungal spores and propagules. These natural inoculants may exhibit seasonal patterns, with the highest numbers of sporulating taxa observed in late summer and early autumn in temperate and boreal forests, e.g. [26–28]. The prior presence of fungal endophytes can influence pathogen–host interactions. As a result, invading pathogens may show altered abilities to form necroses (expanding zones of dying and dead tissue), which are commonly used as bioassays in artificial inoculation experiments as a proxy for the resistance of the tree genotypes, e.g. [29–31]. However, the potential role of fungal microbiomes in shaping necrotic lesion development, both in general and specifically within the Scots pine–*D. sapinea* pathosystem, remains insufficiently understood.

In this study, we hypothesized that the fungal community within Scots pine shoots influences the extent of necrotic lesions caused by *D. sapinea*, and that a well-established endophytic fungal community results in smaller necrotic lesions. To test this hypothesis, we enclosed pine shoots in mesh bags to limit aerial fungal inoculum. We then performed artificial inoculations in the laboratory to compare *D. sapinea*-induced necrosis between shoots that had been covered with bags and those left unbagged. The findings are examined in terms of their ecological significance and their implications for practical tree breeding and forest management.

## Methods

### Test site and field experiment

**Fig. 1:**
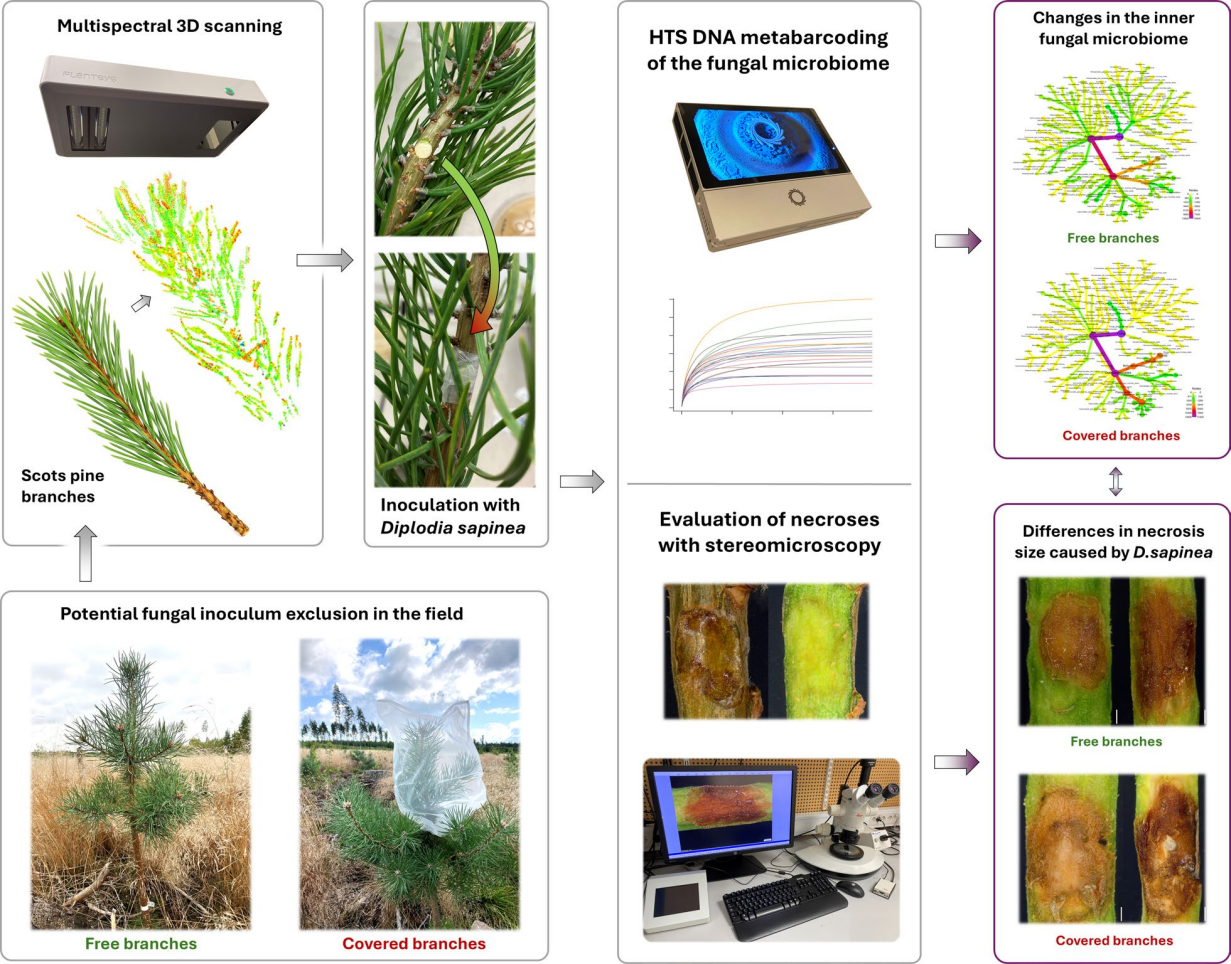
Graphical overview of the study

In the present study, potential fungal inoculum of young Scots pine shoots was partially excluded artificially, and after collection, the shoots were inoculated with *D. sapinea*, assessed under laboratory conditions, and evaluated for necrotic lesions and fungal microbiome composition (Fig. 1).

Our field test site is located in Åryd (near Växjö, Sweden; N 56° 51′ 38.35″ E 14° 59′ 21.90″), where 200 two-year-old Scots pine seedlings (ca 50–100 cm high) were planted in April 2023. The seedlings were grown in pots at Ekebo experimental plant nursery and are of Gotthardsberg seed orchard origin, a common commercial FRM (Forest reproductive material) in Southern Sweden. For this study, we selected individuals that had been maintained under similar conditions in the nursery (regularly watered and kept outdoors). The seedlings were randomly planted in four blocks. Thirty-two morphologically homogeneous plants with comparable height, shoot architecture, and general vigor, were selected for the experiment. To exclude a significant portion of airborne fungal inoculants that could colonize the shoots, we applied 100 µm mesh bags (60 × 40 cm), a mesh size chosen as a compromise that reduces propagule deposition while maintaining normal airflow and minimizing microclimate alteration. Three to five shoots on half of the selected plants were covered with these bags on 5 August 2024, always leaving at least half of each plant’s shoots free (unbagged). The bags remained on the plants for 80 days. Although 100 µm mesh is not small enough to completely block fungal spores and the 80-day period only spans the peak of airborne inoculant activity [26], this duration was chosen as a practical compromise that allowed measurable differences in propagule exposure to develop while still preserving natural shoot conditions as much as possible.

On 23 October 2024, we collected one 20–35 cm long covered (bagged) and unbagged (free) shoot from each of the 32 plants. By visual inspection, the size and greenness of the 16 covered shoots did not differ markedly from the 16 free shoots, nor did the plant individuals. The shoots were cut on-site and immediately placed in 50 ml Falcon tubes filled with water to maintain moist and constant conditions until further processing in the laboratory. On the same day, each shoot was placed in a 200 ml flask with water and scanned using a PlantEye F600 multispectral 3D scanner (Phenospex, Heerlen, The Netherlands) to verify whether covering with mesh bags caused detectable morpho-physiological differences between previously covered and free shoots prior to the inoculation experiment. The PlantEye system combines laser triangulation–based 3D reconstruction with multispectral reflectance measurements to quantify plant morphological and spectral traits and is widely used for plant phenotyping applications [32, 33].

### Inoculation experiment with Diplodia sapinea

On the day of shoot collection, the shoots were inoculated with *D. sapinea* (culture DS01 from the SLU culture collection, Uppsala), an isolate with known pathogenicity. The internal transcribed spacer (ITS) of the nrDNA and partial translational elongation factor 1-alpha (TEF) region of the strain were amplified and sequenced, following Pintye & Knapp [34], and also mitochondrial small subunit ribosomal DNA (mtSSU) using the primer pairs mrSSU1-mrSSU3R sensu Zoller et al. [35], and were deposited in NCBI GenBank under the accessions PV682573 (ITS), PV683232 (TEF), PV682934 (mtSSU). This isolate was collected from the same seedling as strain L_DsA1 (ITS GenBank acc. no.: MT457611), but from a different needle in a nursery in central Sweden (59°37.9181′ N, 17°30.8624′ E) [36]. The inoculation experiment followed the method of [30]. A few needles were removed from the middle of each shoot, and a ~ 5 mm-diameter area of periderm was removed under sterile conditions. A 5 mm-diameter fungal plug, taken from the actively growing edge of a two-week-old *D. sapinea* colony on malt extract agar (MEA, VWR International), was placed on this spot and sealed with Parafilm. Eight shoots from saplings with covered shoots and eight from plants with free shoots were inoculated with *D. sapinea*. The remaining 16 shoots (eight covered and eight free) were inoculated with MEA media as controls. The 32 inoculated shoots were placed in a growth chamber for three weeks under 10 h light/14 h dark conditions at 20 °C. The shoots were watered and repositioned regularly.

After 21 days, the shoots were scanned again using the multispectral 3D scanner. A ~ 2 cm section containing the inoculated area was cut from each shoot. The periderm, parafilm, and fungal plug were carefully removed under sterile conditions. Each inoculated area was then photographed, and the necrotic area was measured using a Leica MZ125 stereomicroscope equipped with an Olympus SC50 Color Camera and analyzed with Olympus cellSens Dimension imaging software. The necrotic tissues were then cut out. After quick surface sterilization by soaking in 70% ethanol for one minute and rinsing three times with sterile Millipore water, a ~ 1 to 1.5 mm section from the edge of each necrotic lesion was cut into 2–3 pieces under sterile conditions and placed onto MEA for *D. sapinea* re-isolation. The re-isolated fungi were later confirmed via ITS sequencing. The remaining necrotic tissue was placed in sterile 1.5 ml centrifuge tubes and frozen at –20 °C.

### Samples used for microbiome analysis

**Fig. 2:**
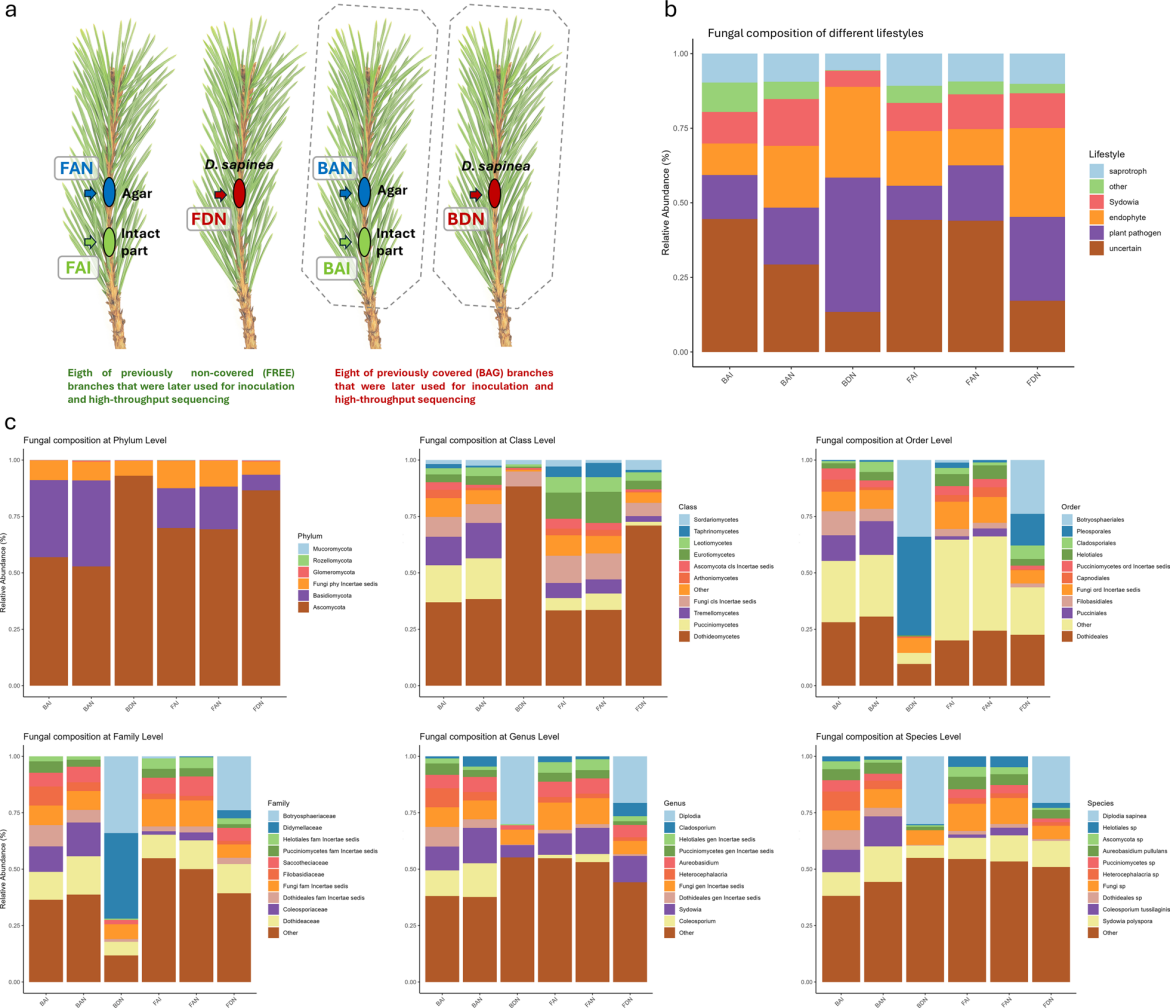
**a** Pictograms of the differently treated shoots visually explaining sample types and groups in the present study. **b–c** Composition of the inner fungal communities of differently treated and inoculated Scots pine shoot samples according to lifestyles (**b**) and on different taxonomic levels (**c**). The 10 most dominant taxa are shown separately, and all the other taxa with lower abundance represents the category ‘other’. Different colors represent the sample groups: Boxplots showing the distribution of relative abundance values for each fungal taxa across the six treatment groups: Intact shoot parts (FAI), agar-inoculated areas (FAN) and *Diplodia sapinea*-inoculated necrotic parts (FDN) of previously free Scots pine shoots, and Intact shoot parts (BAI), agar-inoculated areas (BAN) and *D. sapinea*-inoculated necrotic parts (BDN) of previously covered Scots pine shoots

For molecular analyses, only selected necrotic and control tissues were used. Prior to DNA extraction, the periderm and other outer tissues were removed from the sampled shoot segments, which were then surface sterilized as described above. Consequently, the metabarcoding samples consisted exclusively of internal shoot tissues surrounding the lesion, including necrotic tissue, adjacent cortical tissues, vascular tissues, and underlying secondary xylem. For fungal DNA metabarcoding of the shoot microbiome, 24 of these samples were selected, representing six treatment groups by four replicates (Table S1, Fig. 2a): Intact shoot parts sampled ~ 3 cm below agar-inoculated areas of previously covered (bag-agar-intact, BAI) and free (free-agar-intact, FAI) shoots representing the ‘closest-to-original’ community within the shoots in the experiment; agar-inoculated areas of previously covered (bag-agar-necro, BAN) and free (free-agar-necro, FAN) shoots serving as control samples to check the effect of the inoculation method; and *D. sapinea*-inoculated necrotic parts of previously covered (bag-*Diplodia*-necro, BDN) and free (free-*Diplodia*-necro, FDN) shoots representing the directly affected communities of necrotic lesions caused by *D. sapinea*.

### DNA extraction and PCR amplification

Frozen samples were subsequently lyophilized using a freeze dryer (ScanVac CoolSafe, Labogene, Denmark) after two days in a freezer. The dried samples were roughly ground with an electric grinder and further homogenized using a Retsch Mixer Mill MM 400 (Retsch GmbH, Germany). Total DNA was extracted from ~ 0.5 mL of homogenized tissue using the DNeasy PowerLyzer PowerSoil DNA Isolation Kit (Qiagen, Germany) following the manufacturer’s instructions. The fungal internal transcribed spacer (ITS) region and the 28S (large subunit, LSU) region of the nrDNA was amplified together using commonly used universal primers ITS1F (CTTGGTCATTTAGAGGAAGTAA [37]) and LR5 (TCCTCCGCTTATTGATATGC; [38]). The PCR reactions (50 μl) contained: 1 × Phusion PCR buffer, 0.2 mM dNTPs, 0.5 μM of each primer, 0.02 U Phusion™ High-Fidelity DNA Polymerase (Thermo Fisher Scientific, Lithuania), and 1.25 μl DNA template. PCR was conducted using an XT96 gradient thermal cycler (VWR International GmbH, Germany) with the following program: initial denaturation at 98 °C for 10 min, followed by 35 cycles of 98 °C for 30 s, 52 °C for 30 s, 72 °C for 60 s, and a final extension at 72 °C for 10 min. Each PCR run included negative controls (no-template controls) to monitor potential contamination during amplification, and no amplification was observed in these controls. PCR products were purified using the PureLink PCR Purification Kit (Thermo Fisher Scientific, Lithuania). Amplicon quality was confirmed on a 1.5% agarose gel stained with SYBR Green (Thermo Fisher Scientific, Lithuania). DNA concentrations were quantified with a Qubit™ 4 Fluorometer (Invitrogen, USA) using the Qubit™ dsDNA High Sensitivity Assay Kit (Invitrogen, USA). All 24 samples were diluted to equal DNA concentrations for library preparation.

### Library preparation, HTS sequencing, data processing and analyses

Amplicon libraries of the 24 samples were prepared using the SQK-RBK114.24 rapid barcoding kit (Oxford Nanopore Technologies, ONT) with 50 ng DNA per sample, following the V14 amplicon sequencing protocol. Sequencing was conducted on a FLO-MIN114 flow cell using a MinION MK1C device and MinKNOW software (v24.02.16). Similarly to the pipeline used in Knapp et al*.* [39], Fast5 files were base-called with the high-accuracy model, demultiplexed, and converted to FASTQ format using Guppy v6.1.5. FASTQ files were processed using the EPI2ME workflow (ONT) which was adjusted to fungal amplicon analysis (Fig. S1). Filtering parameters were: read length 300–1500 bp, minimum Q score 12. Taxonomic classification was performed using Kraken2 (v2.1.2) [40, 41] with a custom database built from the UNITE + INSD fungi reference dataset (v21.04.2024) [42], using default k-mer settings (k = 35). The Kraken2 database included fungal ITS and quite often also LSU sequences representing UNITE Species Hypotheses (SH) that ensure the highest possible taxonomic resolution [43]. To refine abundance estimates, a known limitation in long-read datasets [41], Bracken v2.6.2 [44] was used with a read length threshold of 1000 bp and a minimum of 10 reads per taxon. Total abundance and total richness metrics were calculated from the original sequence data, while all subsequent statistical analyses were performed using a rarefied species count Table (4131 sequences per sample; Table S1) obtained by random subsampling of all samples to a uniform sequencing depth. Raw sequence reads are available in the NCBI Sequence Read Archive under BioProject ID PRJNA1266744.

To connect explanatory taxon-based variables with necrotic and healthy tissues and coverage of the pine shoots, raw read counts were first standardized using a centered log-ratio (CLR) transformation to account for the compositional structure of the data. A Random Forest classifier [45] was then trained on the CLR-transformed abundance table derived from the Bracken2 output to evaluate classification accuracy and identify the most informative taxa. Model stability was assessed by repeating the Random Forest procedure 100 times using bootstrapped sample sets. For each taxon, Wilcoxon rank-sum tests were applied to compare CLR abundances between the sample group pairs, and effect sizes were quantified using rank-biserial correlations, followed by false-discovery-rate (FDR) correction. Finally, LASSO-penalized logistic regression [46] was used for multivariate fungal biomarker discovery, enabling selection of a minimal set of taxa jointly associated with the two sample group pairs.

Ecological guilds were assigned using FungalTraits [47]. Designations of plant pathogens and saprotrophs without any indication of endophytic nature were based on their primary lifestyles. Fungi were classified as endophytes if any primary, secondary, or lifestyle-related notes indicated endophytic potential. To avoid potential bias in the classification of endophytes and plant pathogens, *Sydowia polyspora*, which exhibits unclear life strategies, particularly in conifers, and accounted for a significant proportion of the reads, was assigned a unique lifestyle category. Less frequent lifestyles represented by only a few taxonomic units, such as ectomycorrhizal, lichenized, and animal-parasitic fungi, were grouped together as ‘other’, while taxa that could not be assigned to any lifestyle category were labeled as ‘uncertain’.

### Statistical analyses

All statistical analyses were conducted in the R environment for statistical computing [48]. Analysis of variance (ANOVA), principal component analysis (PCA), non-metric multidimensional scaling (NMDS), post-hoc tests and t-tests were performed using R packages vegan [49], ggplot2 [50], ggforce [51], gridExtra [52], tidyr and dplyr [53]. Heat trees were generated using the metacodeR package [54].

For pairwise comparisons of the variables derived from multispectral analyses, t-tests with Benjamini–Hochberg correction, one-way ANOVA with Tukey’s HSD tests and PCA were applied.

Rarefied abundance and richness were compared using ANOVA followed by Tukey’s HSD test, and the results were visualized as boxplots using the ggplot2 package in R [50].

Compositional normalization was carried out using base R functions to compute CLR transformations. Random Forest models were implemented with the ranger package. Non-parametric statistics, including Wilcoxon rank-sum tests and rank-biserial effect sizes, were performed using rstatix [55]. Penalized logistic regression for multivariate biomarker selection was conducted using glmnet [56], following the log-ratio LASSO framework [46]. Heatmaps for explanatory variable visualization were generated with pheatmap [57].

Differences in fungal community composition among samples were visualized using non-metric multidimensional scaling (NMDS) based on Bray–Curtis dissimilarity, applied to a Hellinger-transformed abundance table. The ordination was carried out with the metaMDS function from the vegan R package [49]. A supplementary table containing the sample groups (e.g. FAI, BAI) was used as a secondary matrix. To quantify the variation explained by these groups, permutational multivariate analysis of variance (PerMANOVA) was conducted using the adonis function in vegan.

## Results

### Multispectral 3D analyses

Multispectral analyses performed prior to the in vitro inoculation experiments on the shoots used for fungal microbiome analysis showed no significant differences between previously covered and free shoots (Fig. S2), indicating that the bagging treatment did not cause detectable morpho-physiological differences before the inoculation experiment. Based on 64 default morphological and spectral traits, including average values such as the Normalized Pigment Chlorophyll Index (NPCI), Normalized Difference Vegetation Index (NDVI), and the intervals of several measured indices, neither principal component analysis (PCA) nor pairwise comparisons revealed significant differences. Thus, we assume there were no major physiological differences between the covered and free shoots prior to inoculation (Fig. S2).

### Inoculation experiment, necrotic lesions

**Fig. 3:**
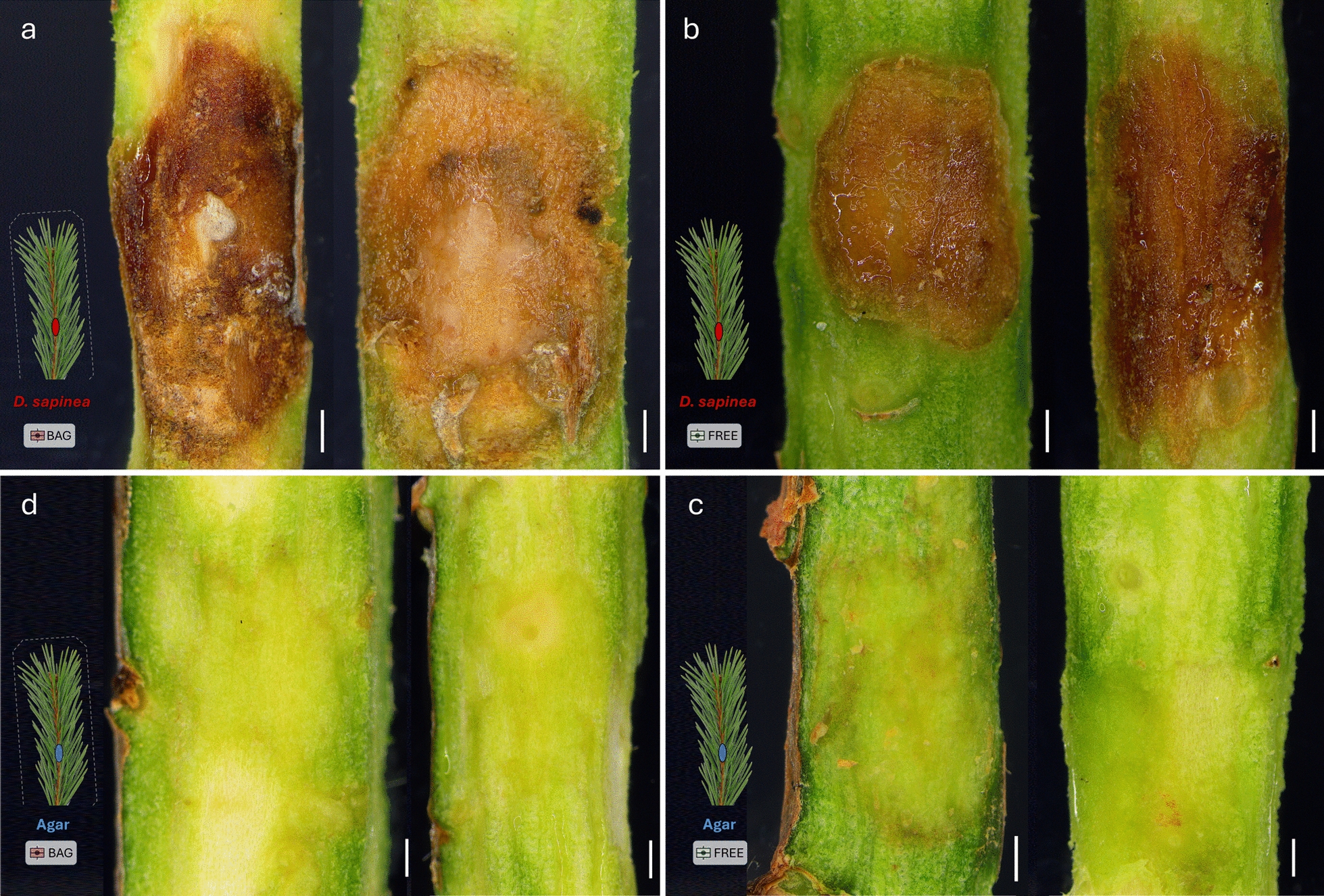
Characteristics of the necrosis and tissues on representative shoots of *Pinus sylvestris* after precise removal of periderm, following inoculation of covered and free shoots by *Diplodia sapinea* and agar plugs. **a** Necrotic tissues of the *D. sapinea*-inoculated previously covered shoots Pin22 and Pin 23, **b** Necrotic tissues of the *D. sapinea*-inoculated previously non-covered shoots Pin09 and Pin10. **c** Tissues at the agar-inoculation area of previously covered control shoots Pin17 and Pin18, **d** Tissues at the agar-inoculation area of previously non-covered control shoots Pin07 and Pin05. The pictograms at the left corners show the cover- and inoculation status of the given shoots. Scale bars = 1 mm

**Fig. 4:**
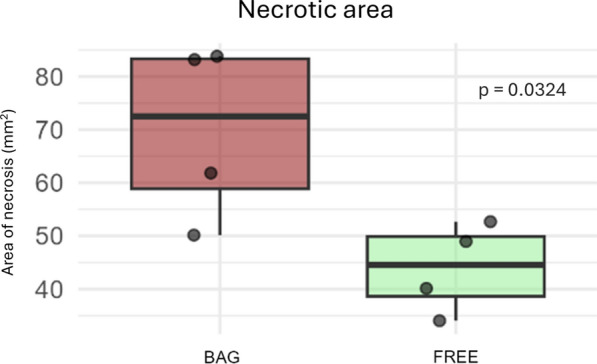
Necrotic area in *Pinus sylvestris* shoots previously covered with mesh bags (BAG) or left uncovered (FREE) and inoculated with *Diplodia sapinea*. Box plots show necrotic area measured in the samples used for microbiome analysis. P values indicate t-tests with Benjamini–Hochberg correction (Table S1)

After the in vitro inoculation tests, no necrosis was observed in any control shoots inoculated with MEA media plugs (Fig. 3c, d). In contrast, shoots inoculated with *D. sapinea* developed necrotic lesions averaging approximately 57 mm^2^ on the sides of the shoots (Fig. 3a, b). The necrotic area was significantly larger (*p* = 0.0327) in previously covered shoots compared to free ones (Fig. 4). This pattern was further supported by differences in lesion perimeter and remained significant when additional samples not designated for microbiome analysis were included, showing similar differences in necrotic area and perimeter (Fig. S3). The pathogen was successfully re-isolated from all but one of the inoculated samples; in that case, two *Sydowia polyspora* cultures emerged instead. Therefore, we assume that *D. sapinea* was present in the necrotic tissues of the inoculated shoots.

### Fungal microbiome analyses

**Fig. 5:**
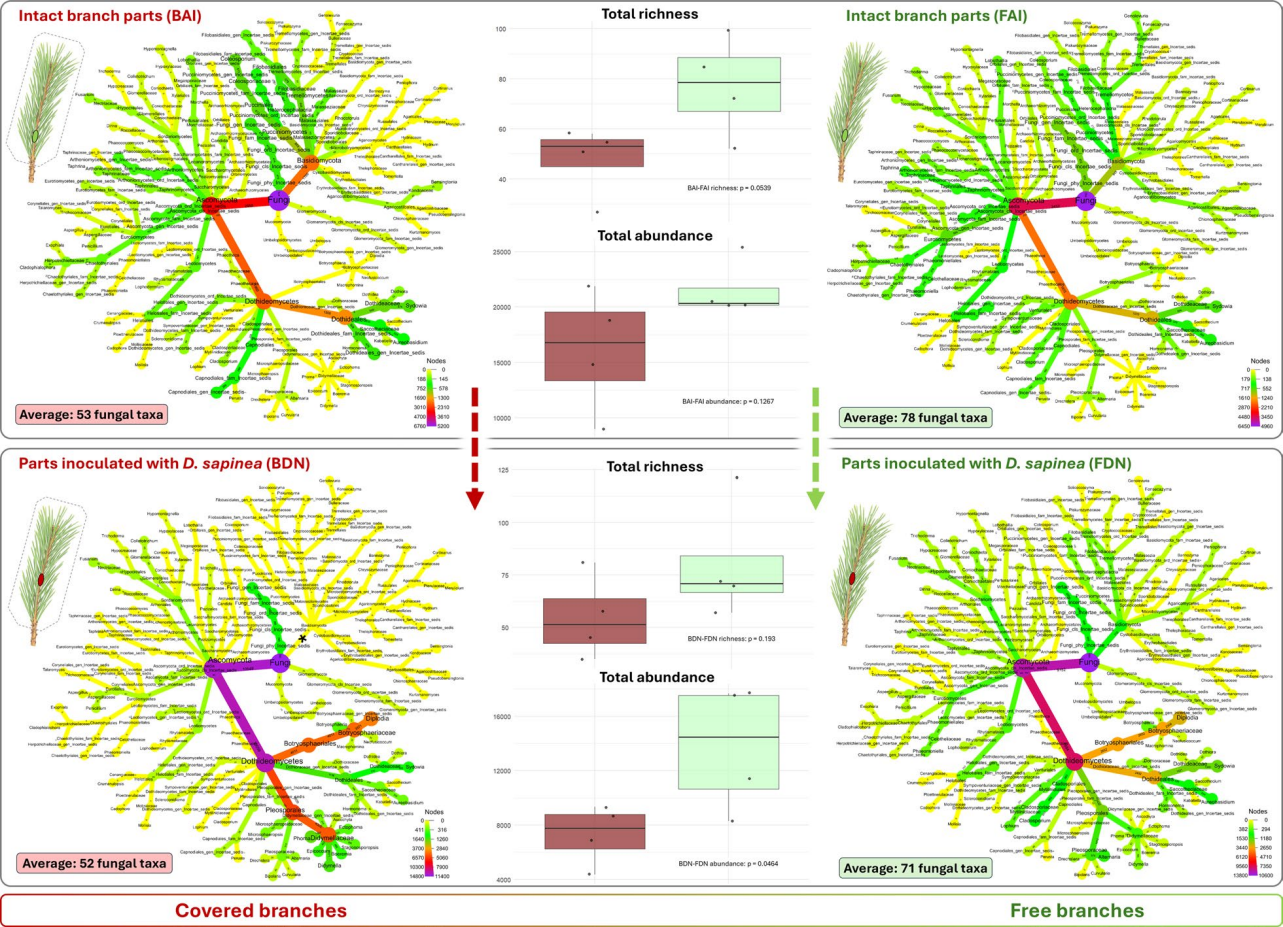
Visualized fungal community structure, total richness and total abundance of four treatment groups: Intact shoot parts (FAI), and *Diplodia sapinea*-inoculated necrotic parts (FDN) of previously free Scots pine shoots (labelled with green), and Intact shoot parts (BAI), and *D. sapinea*-inoculated necrotic parts (BDN) of previously covered Scots pine shoots (labelled with dark red). Each node represents a taxon from kingdom to genera, and only the taxa representing more than 0.05% of the total fungal reads were visualized in the heat tree. Tree topology is the same for every sample, whereas color of the nodes goes from purple (100% relative abundance), through red, orange and green to yellow (0% relative abundance) according to abundance of the taxa in each sample. Small numbers on the edges give abundance of the taxa in read numbers, which follow the given edge. The asterisk marks the branch of phylum Basidiomycota. Pictograms of shoots at heat trees show the cover- and inoculation status of the given samples. Box plots show the total richness and total abundance of intact, and *D. sapinea*-inoculated parts previously covered (BAG) and free (FREE) shoots of Scots pine. At the plots, p values refer to Tukey’s HSD tests (Table S1)

We analyzed the fungal microbiome of 24 Scots pine shoot samples representing intact parts of previously covered and free shoots, as well as portions of these shoots inoculated with either *D. sapinea* or MEA (agar) as a control. After ONT sequencing, the EPI2ME pipeline yielded a total of 362,849 sequences, which were rarefied to 4131 reads per sample (Fig. S4). These sequences represented 347 taxa (Fig. 5, Table S1). Among these, the most dominant fungal taxon was the inoculant pathogen *D. sapinea*, which was exclusively present in the shoots inoculated with it. The second most abundant was *S. polyspora*, which dominated the native fungal community in all samples. The vast majority of identified fungal taxa belonged to the phylum Ascomycota. Samples of the six treatment groups included several dominant taxa across various taxonomic levels, and featured typical lifestyles of plant-associated fungi, including plant pathogens, saprobes, and endophytes (Fig. 2, Table S1).

**Fig. 6:**
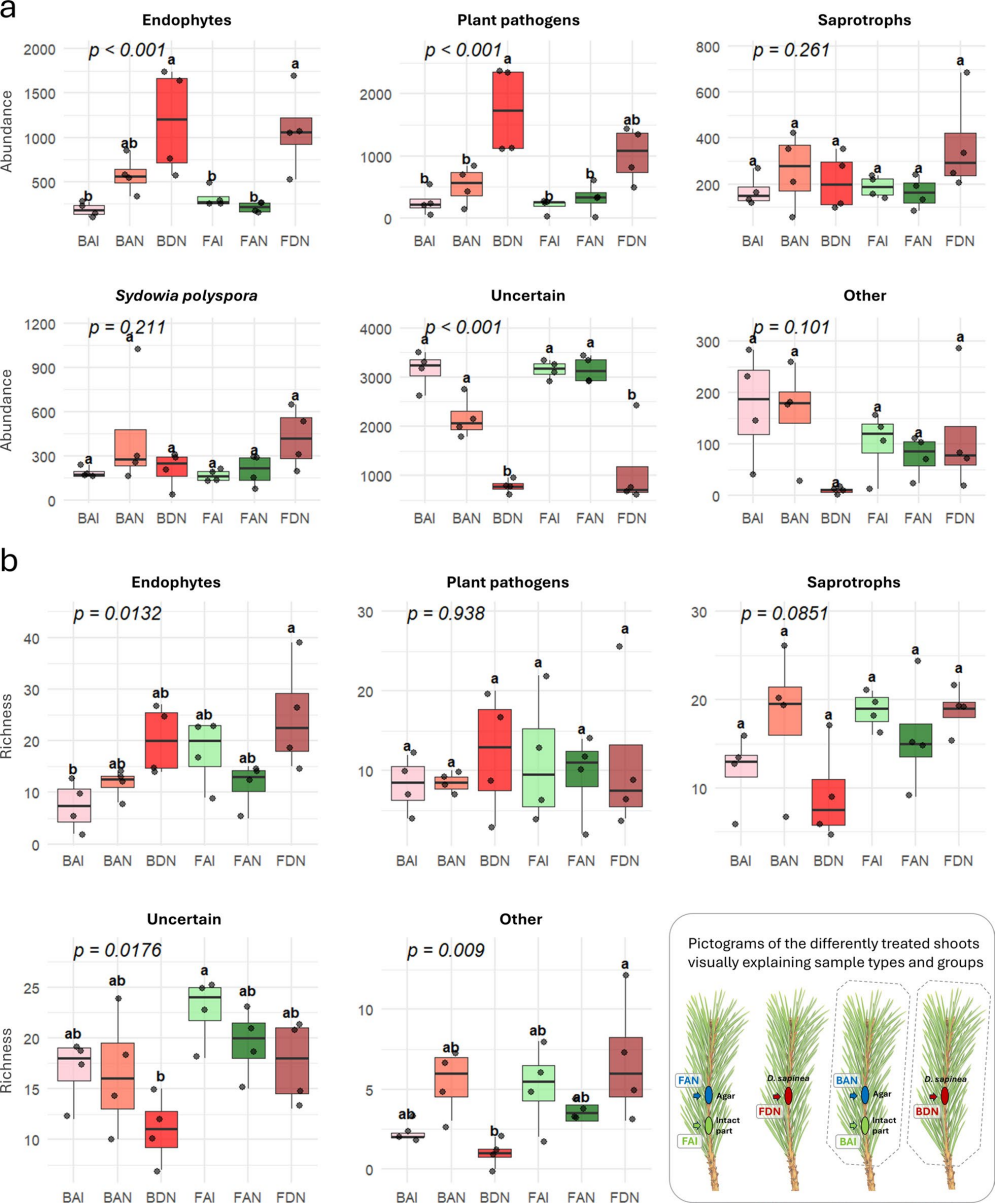
Boxplots showing fungal abundance and richness across treatment groups and lifestyle categories based on rarefied read counts. Different colors represent the sample groups: Intact shoot parts below the agar-inoculated covered (bag-agar-intact, BAI) and free shoots (free-agar-intact, FAI), agar-inoculated shoot parts of covered (bag-agar-necro, BAN) and free shoots (free-agar-necro, FAN), and parts inoculated with *D. sapinea* in the case of covered (bag-*Diplodia*-necro, BDN) and free shoots (free-*Diplodia*-necro, FDN). Means were compared using ANOVA and Tukey’s HSD tests, with letters denoting significant (*p* < 0.05) differences within each boxplot. Statistical summaries are provided in Table S1. A pictogram panel in the lower right corner illustrates the treatment groups and corresponding sample abbreviations

Comparisons of fungal rarefied abundance and richness among fungi with different lifestyles revealed significant differences among treatment groups (Fig. 6). The abundance of endophytes and plant pathogens followed a similar pattern: both were less abundant in intact and control samples and increased after inoculation. However, a significant increase in pathogen abundance was observed only in the covered *D. sapinea*-inoculated samples, not in the corresponding free shoot samples. In contrast, *S. polyspora*, saprotrophs, and fungi with other lifestyles did not show significant differences. Fungi that could not be assigned to specific lifestyles showed decreased abundance in *D. sapinea*-inoculated samples. Richness across fungal lifestyles showed no significant differences among the sample pairs, except for fungi with other lifestyles, which were more diverse in the free *D. sapinea*-inoculated shoots compared to the previously covered ones (Fig. 6). Although the total richness was not significantly different between FAI and BAI (*p* = 0.0539), the free shoot samples (FAI) contained an average of 78 fungal taxa, compared to 53 taxa on average in the covered shoot samples (BAI), indicating substantial differences (Fig. 5). There was no significant difference between the intact and agar-inoculated groups (FAI-FAN and BAI-BAN) based on major taxonomic groups (Fig. 6). Similarly, no differences were observed in abundance and richness of different fungal lifestyles (Figs. 6 and S5).

We found significant differences in the relative abundance of fungal taxa among the sample groups. The heat trees clearly show shifts in community structure, especially between intact shoots and those inoculated with *D. sapinea* in the case of covered shoots (Figs. 5, S6). The free intact shoots showed higher abundance for certain lower fungal taxa such as the genera *Exophiala* and *Genolevuria* and a *Trichoderma* species, *T. asperellum* (Fig. S7). Some of the higher-level taxa, such as the dominant classes Sordariomycetes and Leotiomycetes were similarly distributed across groups (Fig. S7). In contrast, other higher-rank taxa showed significant differences, mainly when comparing intact and *D. sapinea*-inoculated samples in the case of covered shoots. We found differences in the whole phylum Ascomycota and within the order Pleosporales (class Dothideomycetes), with a higher abundance in BDN compared to BAI, while no difference was observed between FAI and FDN. Significant differences were also found in the phylum Basidiomycota, which was higher in BDN than in BAI, with no difference between FAI and FDN (Fig. S7). To assess the overall similarity among samples, non-metric multidimensional scaling (NMDS) based on Bray–Curtis distances was performed. The resulting fungal community structures showed separated groupings (Fig. S5). Both intact tissues and agar-inoculated parts exhibited separation between covered and non-covered shoots, corresponding to differences in fungal lifestyles. In the case of endophytic fungi, both FAI and BAI groups were also separated.

**Fig. 7:**
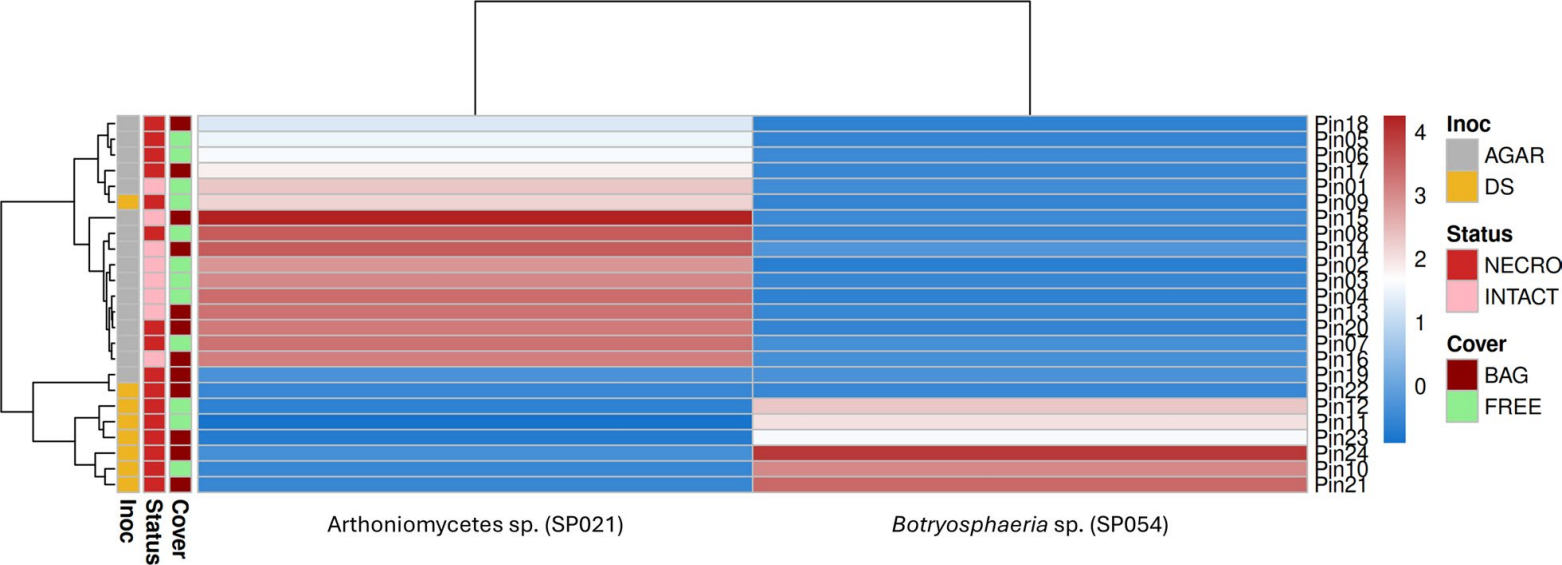
Heatmap of necrosis- and healthy tissue-associated fungal marker taxa in Scots pine shoot woody tissues. The heatmap shows CLR-transformed abundances of the top two fungal taxa identified by LASSO logistic regression as the strongest predictors of tissue types (*Diplodia sapinea*-associated necrotic, and intact woody tissues): *Arthoniomycetes* sp. (SP021), which is depleted in DS-inoculated tissues, and *Botryosphaeria* sp. (SP054, identified as *B. stevensii* during the taxonomic assignment), which is enriched in *D. sapinea*-inoculated tissues. CLR abundance values are shown on a continuous colour scale ranging from the lowest relative abundance (deep blue) to highest relative abundance (deep red), reflecting the log-ratio–based compositional normalization of the dataset. Rows represent individual samples highlighted based on presence or absence of mesh-bag coverage (dark red = BAG, light green = FREE), Tissue status (red = NECRO, pink = INTACT), and Inoculation material (grey = AGAR, yellow = DS). Clustering was performed using average linkage on CLR-transformed data

Using the Random Forest classification and LASSO-penalized logistic regression, we tested whether the use of mesh-bags influenced fungal community composition (after removal of the artificially added *D. sapinea*), and the CLR-LASSO regression selected no taxa at the optimal penalty (λmin = 0.2448), retaining only the intercept (0 non-zero coefficients). The model discrimination was identical to random expectation (AUC = 0.50), demonstrating that the use of bags did not result in unbalanced community composition with the significant presence or absence of any taxonomic groups. However, we could identify two fungal markers as the strongest predictors distinguishing necrotic from healthy tissues (Fig. 7): Arthoniomycetes sp. (SP021) and a *Botryosphaeria* species (SP054) assigned by the Kraken2 taxonomic classification to *B. stevensii*. Arthoniomycetes sp. was significantly depleted in *D. sapinea*-inoculated samples (rank-biserial correlation r_rb = –0.92 in DS direction; LASSO coefficient = –0.0667) exhibiting a strong negative association with necrosis displaying the highest effect size, whereas the *Botryosphaeria* species was significantly enriched (rank-biserial correlation r_rb =  + 0.69; LASSO coefficient =  + 0.138) showing positive association with necrosis. These taxa form a clear bidirectional microbial signature of *D. sapinea* inoculation. These two taxa may therefore represent potential opposite-direction biomarkers, with the *Botryosphaeria* species enriched in necrotic tissues and Arthoniomycetes sp. depleted, forming a potential discriminatory signature between tissue states (Fig. 7).

## Discussion

### Fungal microbiome of Scots pine shoots

In this study, we used third-generation DNA metabarcoding to examine fungal communities in young Scots pine shoots. Although ONT-based fungal DNA metabarcoding currently lacks a unified processing pipeline and in-built accurate fungal reference databases, an increasing number of studies have begun to apply it [58–62], several of which using the Kraken2 classification method [63–66]. Here, we applied the EPI2ME workflow using the Kraken2/Bracken pipeline with a custom-made UNITE + INSD fungal reference dataset for the samples, and the rarefaction curves showed clear saturation, indicating that sequencing depth had reached the expected plateau (Fig. S4).

In recent years, several studies have investigated the fungal microbiome of Scots pine shoots using various fungal DNA metabarcoding techniques and key fungal community members have been identified across different countries and regions [17, 19, 67–70]. In these studies, high-throughput sequencing of mainly the ITS2 region has revealed that Scots pine shoots host diverse fungal communities, including both endophytes and potential pathogens, often dominated by certain lineages such as *D. sapinea*, *S. polyspora*, or other ascomycetous taxa. Variation in fungal community composition seems to be primarily influenced by geography and site-specific differences [19]. Interestingly, although *Diplodia* spp. is considered a common endophyte in pine trees, not a single read of this fungus was found in the shoots prior to inoculation in our study. It was also absent in neighboring naturally grown > 10-year-old Scots pine trees (Knapp et al*.*, unpublished data). *Diplodia sapinea* is commonly found as a member of the fungal microbiome of Scots pine trees [17, 22, 68, 71]. In a recent study [19], *D. sapinea* was found in both healthy and symptomatic twigs of Scots pine, however, it was far more abundant in diseased branches [19]. The absence of *D. sapinea* in our examined pine shoots, despite its frequent detection in Scots pine across Europe, may reflect strong spatiotemporal variability in airborne inoculum, with sporulation peaks occurring primarily during warm, dry periods and varying between sites and years [20, 28]. The location of our field site in southern Sweden may also currently experience low natural inoculum pressure, consistent with the absence of *D. sapinea* in nearby mature trees. In addition, endophytic colonization by *D. sapinea* often reflects latent infection of older or stressed tissues, whereas our sampled shoots were young and healthy shoots in which *Diplodia* colonization is likely less common or more transient [17, 22]. Furthermore, microbial assembly in newly developing shoots is strongly shaped by priority effects and early-colonizer competition [72, 73], and dominant ascomycetes such as *Sydowia polyspora* may effectively exclude or outcompete *D. sapinea* during early establishment, particularly under low propagule pressure. This absence likely strengthens our interpretation by providing a clean baseline from which the effects of the experimentally introduced inoculum can be clearly resolved.

### Fungal microbiome of the inoculated covered and free shoots

The fungal communities were differently established, with slightly lower total fungal abundance and differences in the distribution of certain taxa and lifestyles in the covered shoots. Due to the lack of significant differences in the lifestyle, taxonomic structure, or richness of fungal communities between the intact and agar-inoculated groups in both covered and free shoots, we conclude that the differences in fungal communities interacting with *D. sapinea* reflect the effect of the pathogen itself rather than the inoculation technique. Therefore, we suggest that the observed taxonomic compositional changes, such as the increase in pleosporalean taxa and the notable absence of basidiomycetes, as well as changes in lifestyle groups, are due to the presence and activity of *D. sapinea* during necrosis formation. Although we found differences in their fungal communities, intact parts of the free and covered shoots differed only in a few specific taxa, while the overall community structure and abundance had changed, suggesting that lower aerial inoculum levels slowed or constrained endophyte establishment rather than altering the overall functional composition of the community. One of the taxa with differing abundance was the genus *Exophiala*, a general endophyte of plants, including conifers [74], which primarily colonizes roots and can be significantly more abundant in healthy plant tissues [75].

Our results highlighted that the fungal composition of the intact part of the shoots, considered to reflect the initial community, differed only slightly. However, the changes following inoculation with *D. sapinea* were in previously covered shoots more pronounced than in the free shoots. Shoots with propagule exclusion exhibited much greater differences in microbiome composition compared to the unbagged ones. The artificially altered microbiome, potentially due to reduced competition and other factors, was associated with a significant increase in pathogen abundance, involving not only the inoculant fungus but also other pathogens. This could also result in the reduction of less pathogenic and less competitive community members, such as the basidiomycetous yeasts that can play a role as biocontrol of plant diseases [76], but which may also remain undetected following fungal–fungal competition [77]. For instance, the complete absence of the phylum Basidiomycota, which is generally represented by saprobic but occasionally endophytic yeasts in Scots pine branch and wood tissues, in *D. sapinea*-inoculated, previously covered shoots is significant. This may reflect the sensitivity of basidiomycetous yeasts to pathogen-driven tissue changes and their reduced competitiveness in simplified microbiomes. Another noteworthy observation is the induced presence of pleosporalean taxa with diverse lifestyles in diseased tissues. We did not find clear similarities in the relative abundance of specific taxa when compared with other studies. For example, the order *Phaeothecales*, which was suggested to be antagonistic in *D. sapinea*-infected Scots pine shoots [19], was not represented by a single read in any of our samples.

When applying random forest-based prediction to identify explanatory taxa of the tissue type, we found that the class Arthoniomycetes or certain taxa there may represent an interesting candidate for further investigation in larger-scale studies due to its potential biomarker-like association with *Diplodia*-infected tissues. Arthoniomycetes comprises mostly lichenized fungi [78], however, their contribution as endophytes cannot be ruled out. This taxon and related OTUs have already been found within pine tree tissues as dominant members of the fungal microbiome in different geographical regions [79, 80] including Sweden where Millberg et al. [79] found Arthoniomycetes sp. representing the largest and most abundant OTU in Scots pine needles. The relative abundance of this OTU in needles exhibiting disease symptoms was found to be increased with needle age suggesting that certain members of the Arthoniomycetes may function as latent saprotrophs that proliferate as needle tissues mature or begin to senesce, which could explain their depletion in actively necrotizing, *Diplodia*-infected tissues. The *Diplodia*-predictor status of another Botryosphaerialean taxon may not be surprising, given the potential co-occurrence and co-infection of multiple *Botryosphaeriaceae* species [81, 82]. Although this taxon in the genus *Botryosphaeria* has been assigned to the species *B. stevensii* (anamorph: *D. mutila* [83]), it may be the result of methodological or reference database limitations. While the species occurs in the region of our study site, Botryosphaerialean OTUs are often present in the pine fungal microbiome [79], dominant occurrence of this particular species is unlikely in Scots pine. Nevertheless, the predictor model and analytical framework applied in this pilot study may still be suitable for identifying indicative fungal taxa associated with specific tissue types in larger-scale studies with more comprehensive datasets.

Using Random Forest classification for comparison of fungal communities between bagged and unbagged shoots, the CLR-LASSO logistic regression identified no informative predictors indicating performance no better than random. This indicates that no taxa were capable of discriminating between treatments. These findings correlate with the results of comparisons of different taxonomic ranks and on the visualization of the communities with heat-trees, in which no consistent presence–absence patterns of specific taxa were observed. Together, these results indicate that the mesh-bag treatment did not selectively exclude particular fungal propagules and certain taxa; instead, it primarily reduced overall richness and total fungal abundance, supporting the suitability of this approach for controlled inoculation studies.

### The necrosis caused by *D. sapinea*

We found that necrosis size in the covered shoots was significantly larger than in the free ones, which may have important implications. For instance, in nurseries and breeding programs, robust and simple bioassays are required during early resistance screening stages to identify tree genotypes with promising resistance [84]. One of the widely used bioassays is based on the formation of necrosis after artificial inoculation with pathogens [29, 31, 85]. Lesion size is used as a proxy for tree resistance, with smaller lesions indicating more effective resistance mechanisms and higher genetic resistance [86–89]. However, polymicrobial regulation of pathogen-induced lesion formation is known [88, 90], whereby prior fungal or microbial presence can alter the pathogen’s ability to cause necrosis, potentially biasing this proxy. This regulation is driven by the associated microbiome of plants, composed mainly of plant-associated bacteria and fungi [72, 91]. However, these interactions cannot be generalized since microbes may influence the invading pathogen either positively or negatively [92–94]. Fungal communities present prior to infection can affect pathogen behavior through various mechanisms and features [95, 96] including greater diversity and complementary functions [2, 97]. The present findings underline and strengthen the importance of a well-established inner fungal community. Our study suggests that the *D. sapinea-P. sylvestris* pathosystem may represent an example in which pathogen virulence is influenced by the fungal community of the host. While the present study focuses on fungal communities, disease development in this pathosystem is likely shaped by multi-kingdom microbial interactions; integrating bacterial and fungal microbiome analyses in future studies may therefore provide a more comprehensive understanding of disease expression.

Although the number of biological replicates in the present study was limited and variability among samples was relatively high, the observed pattern of larger necrotic lesions in shoots with reduced propagule exposure was consistent across treatments. Therefore, the results should be interpreted as an initial indication of microbiome-mediated modulation of lesion development that merits further investigation in larger-scale studies. Despite these limitations, the findings may still have relevance for breeding programs: if endophytic community structure affects lesion development, then variation in microbiome status may introduce unintended noise or bias into lesion-based resistance screening. Recognizing this source of variation, and considering ways to standardize or account for it, may improve the reliability of early-stage resistance assays, as suggested in other pathosystems where microbial associates modulate host–pathogen interactions. This insight may have future applications in forest management, for example by potentially influencing the mycobiome of seedlings through fungal inoculants to promote beneficial endophyte communities before pathogen exposure [28].

## Conclusion

Our study highlights the possible importance of microbiome legacy as a modulator of disease outcomes in plants. This work provides a useful framework for combining microbial analysis, plant phenotyping, and pathogen testing. Notably, necrotic areas were roughly 50% smaller in shoots associated with a more established fungal community, suggesting a potential microbiome effect on lesion development. Although more studies with greater sample sizes and a wider range of pathogens are needed, our results point to the importance of overall fungal richness rather than single taxa in shaping this response, and future work should test how consistently these microbiome effects influence lesion-based resistance assessments. Our findings support the importance of plant microbiomes in tree health and point to possible applications in tree resistance breeding and forest management, such as using beneficial microbes to improve disease resistance in young trees.

## Supplementary Information

Supplementary Material 1.

Supplementary Material 2.

Supplementary Material 3.

Supplementary Material 4.

Supplementary Material 5.

Supplementary Material 6.

Supplementary Material 7.

Supplementary Material 8.

Supplementary Material 9.

## Data Availability

The sequencing data has been deposited in the NCBI Sequence Read Archive (SRA) under BioProject ID PRJNA1266744. Sequences of three DNA loci of the *Diplodia sapinea* culture DS01 have also been deposited in NCBI GenBank under the accessions PV682573, PV683232, PV682934.
