# Supplementary figures and images for: Microbiome legacy influences necrosis formation in Diplodia sapinea-infected Scots pine shoots
Source: Environ Microbiome. 2026 May 9;21:64. doi: 10.1186/s40793-026-00904-9 (PMC13162409; doi:10.1186/s40793-026-00904-9)

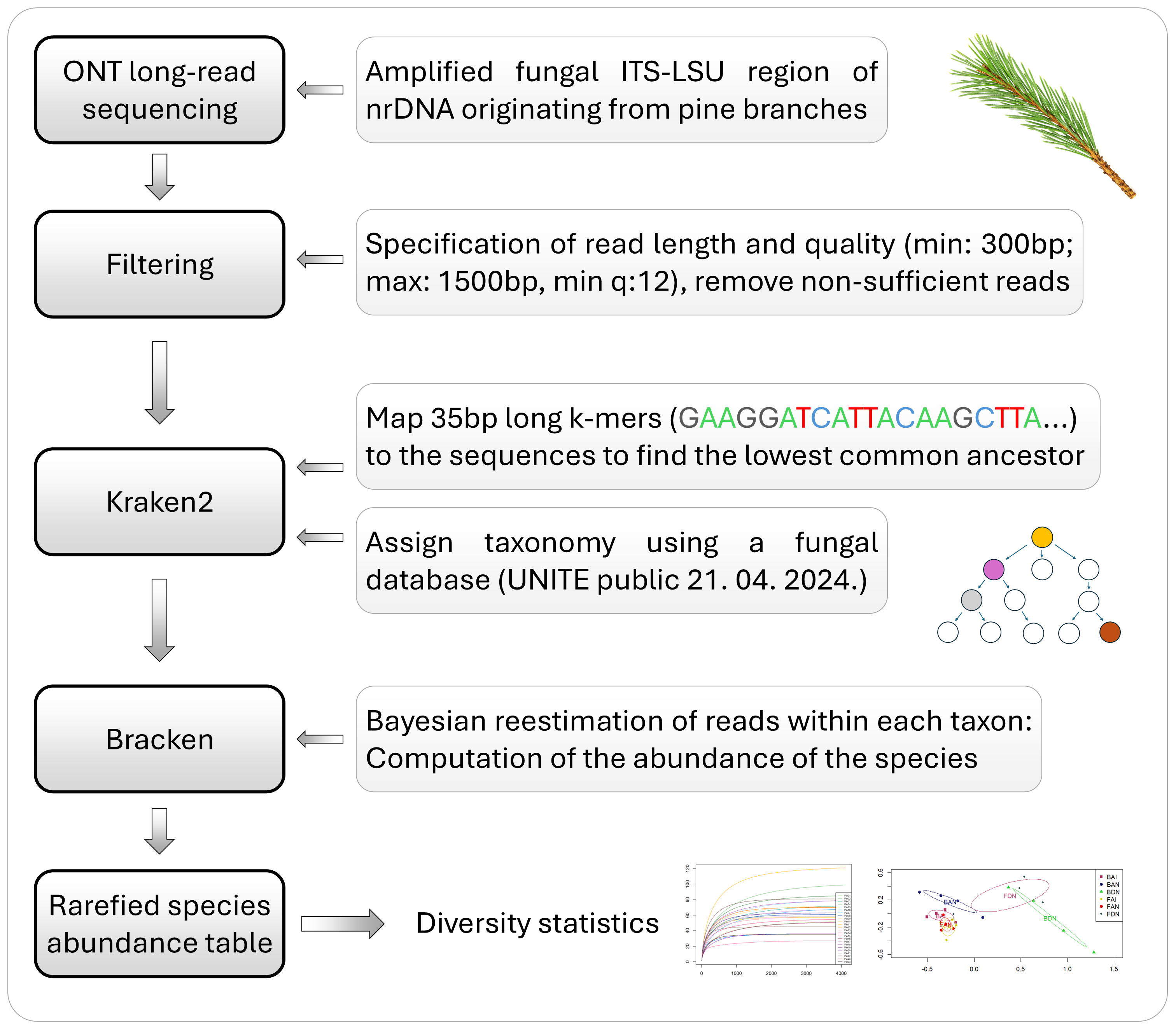

Supplement: Supplementary file 1 — Supplementary Material 1. [file 40793_2026_904_MOESM1_ESM.png]

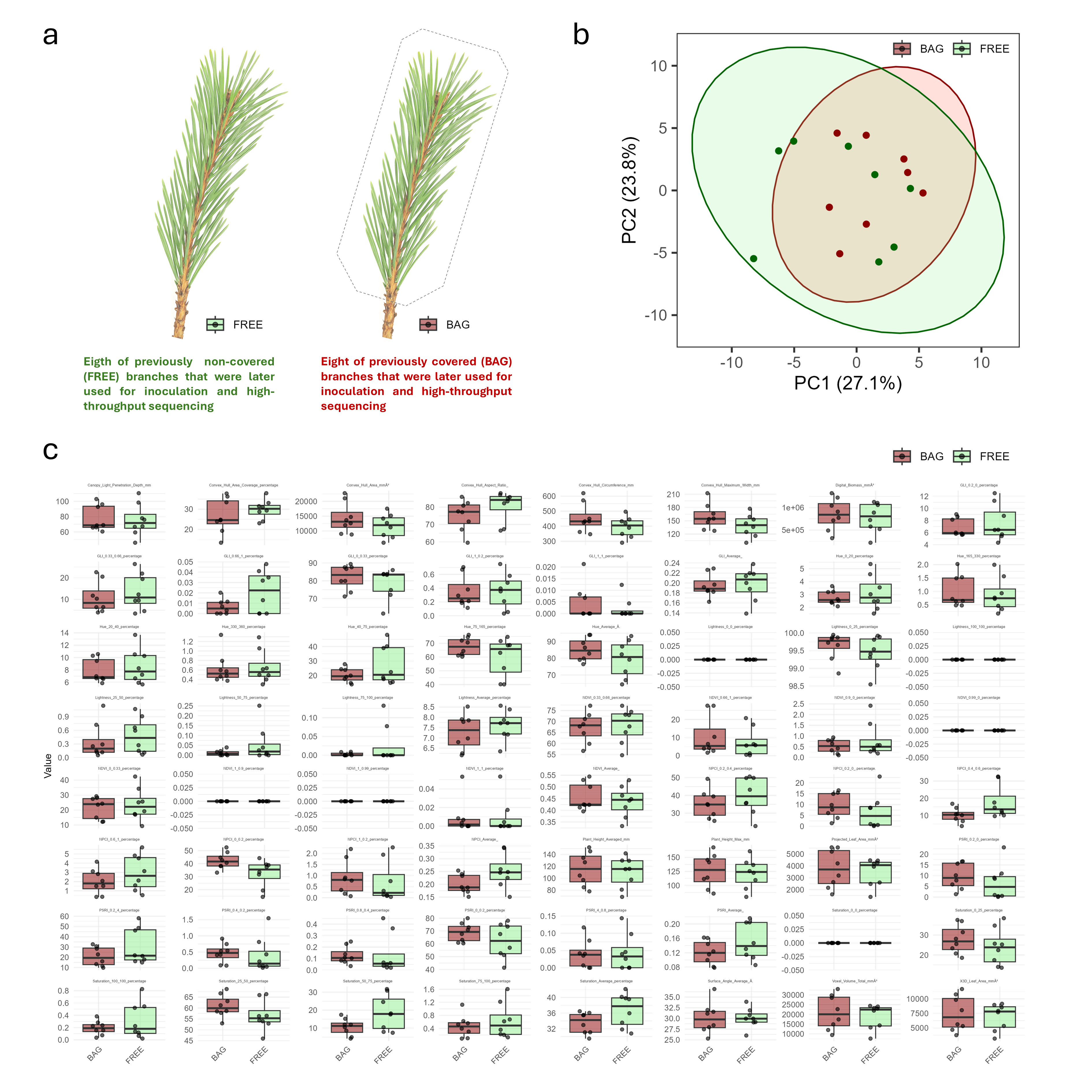

Supplement: Supplementary file 2 — Supplementary Material 2. [file 40793_2026_904_MOESM2_ESM.png]

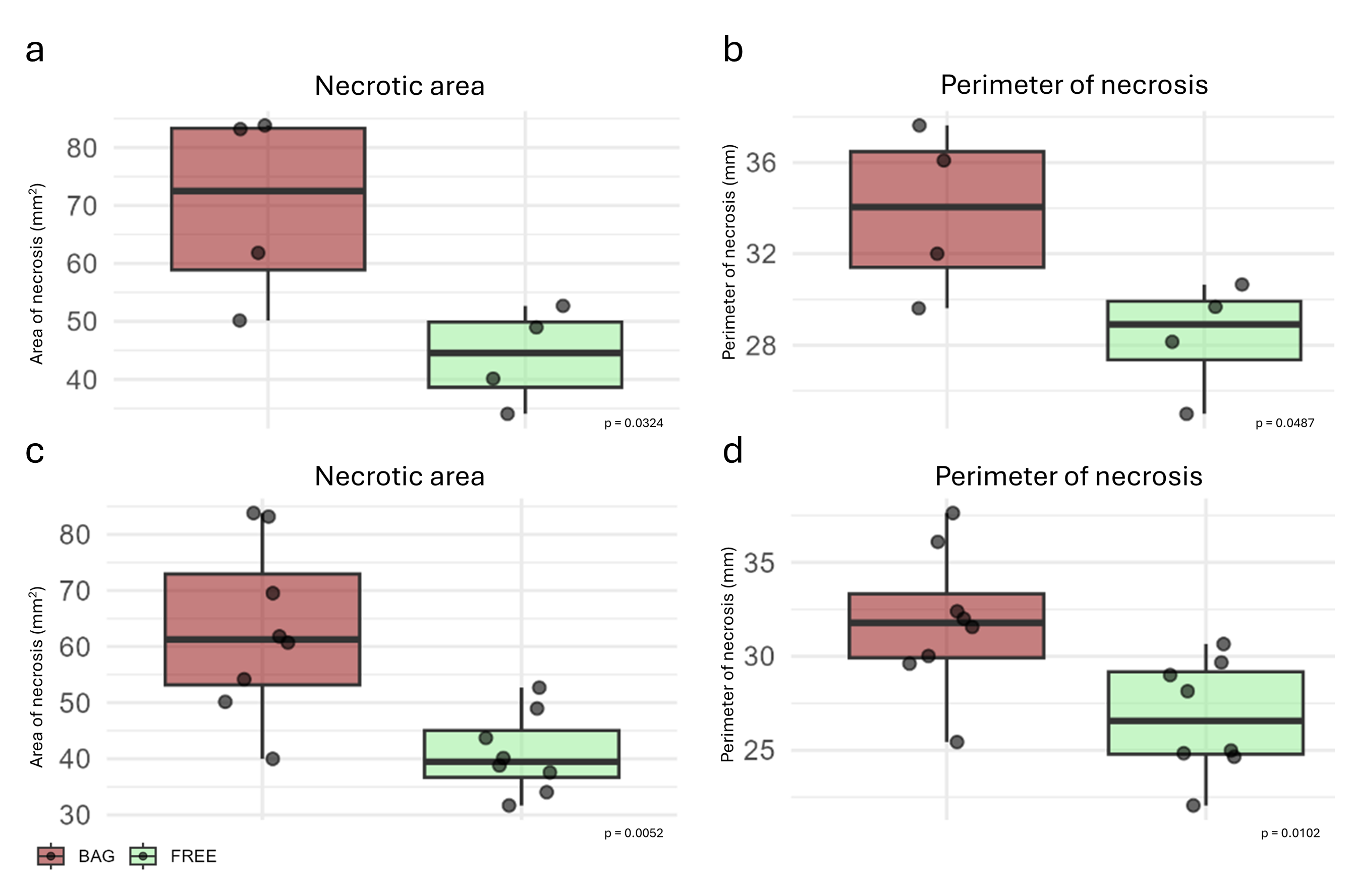

Supplement: Supplementary file 3 — Supplementary Material 3. [file 40793_2026_904_MOESM3_ESM.png]

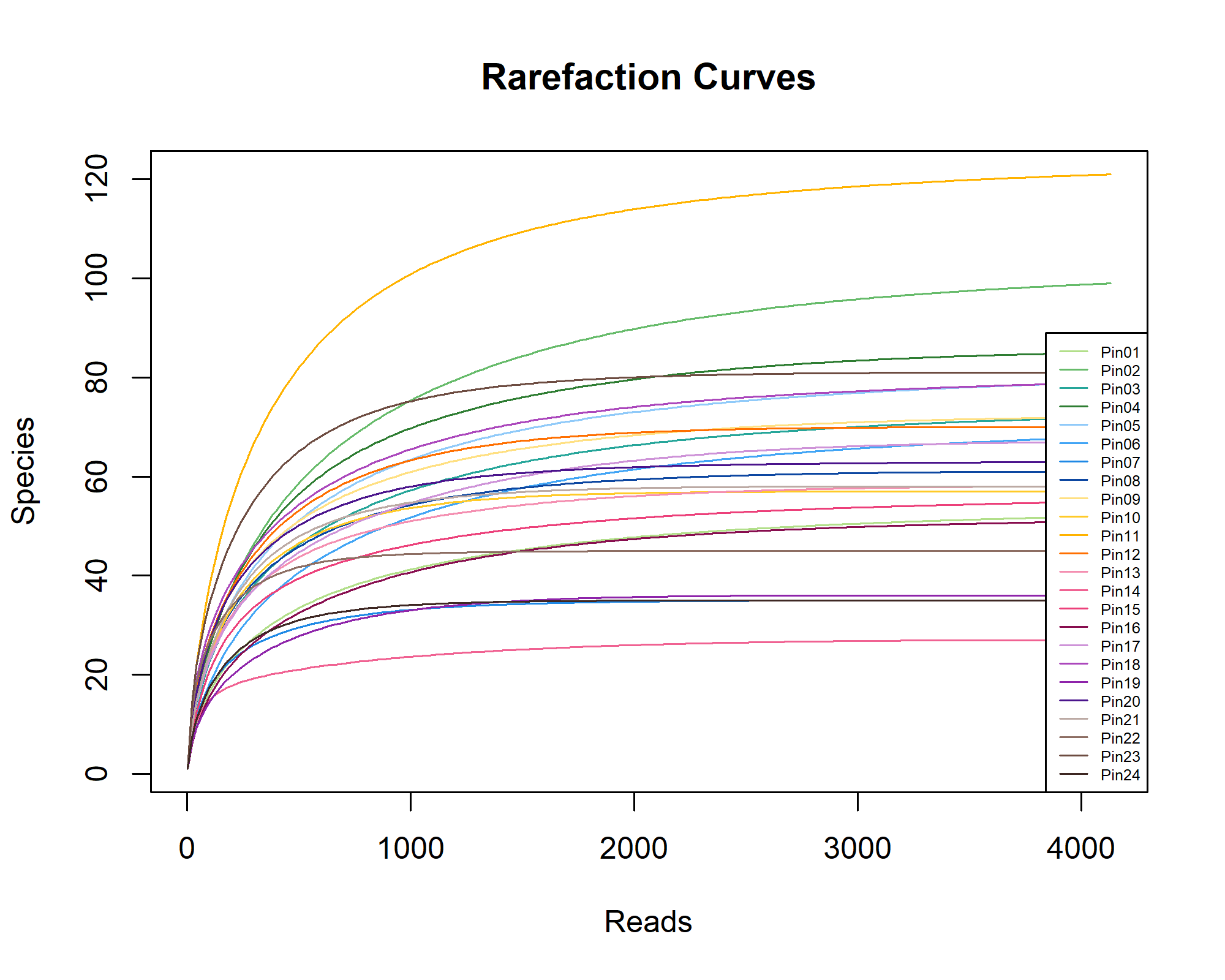

Supplement: Supplementary file 4 — Supplementary Material 4. [file 40793_2026_904_MOESM4_ESM.png]

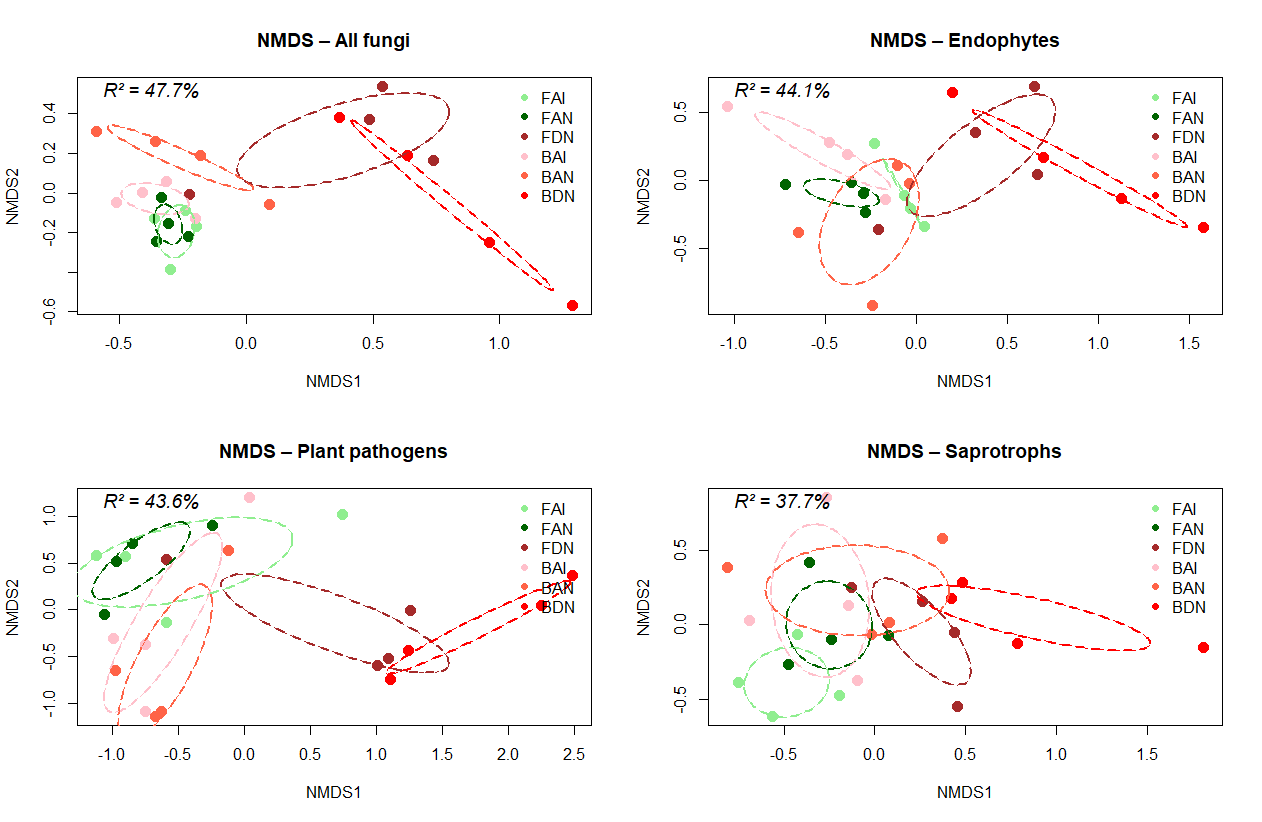

Supplement: Supplementary file 5 — Supplementary Material 5. [file 40793_2026_904_MOESM5_ESM.png]

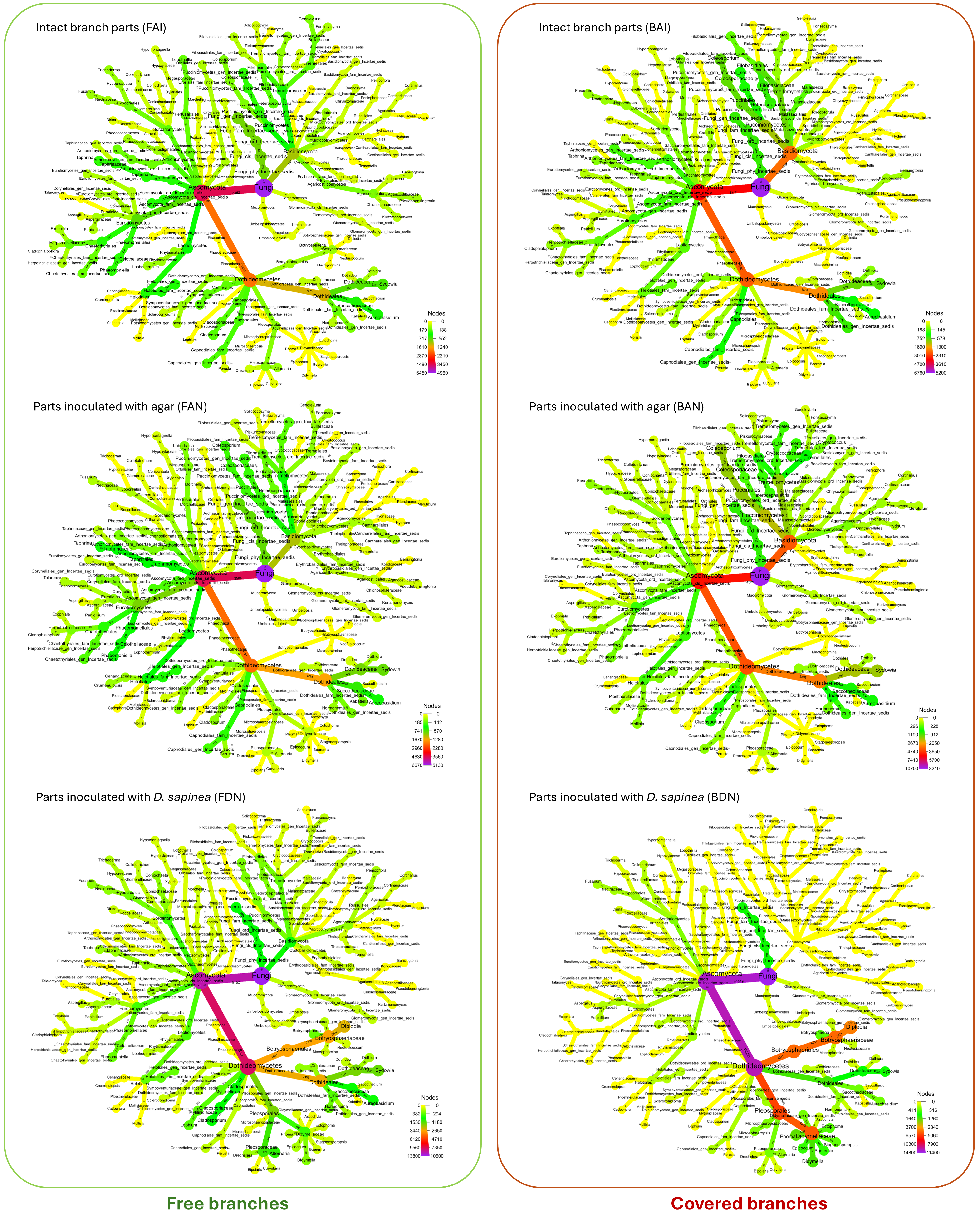

Supplement: Supplementary file 6 — Supplementary Material 6. [file 40793_2026_904_MOESM6_ESM.png]

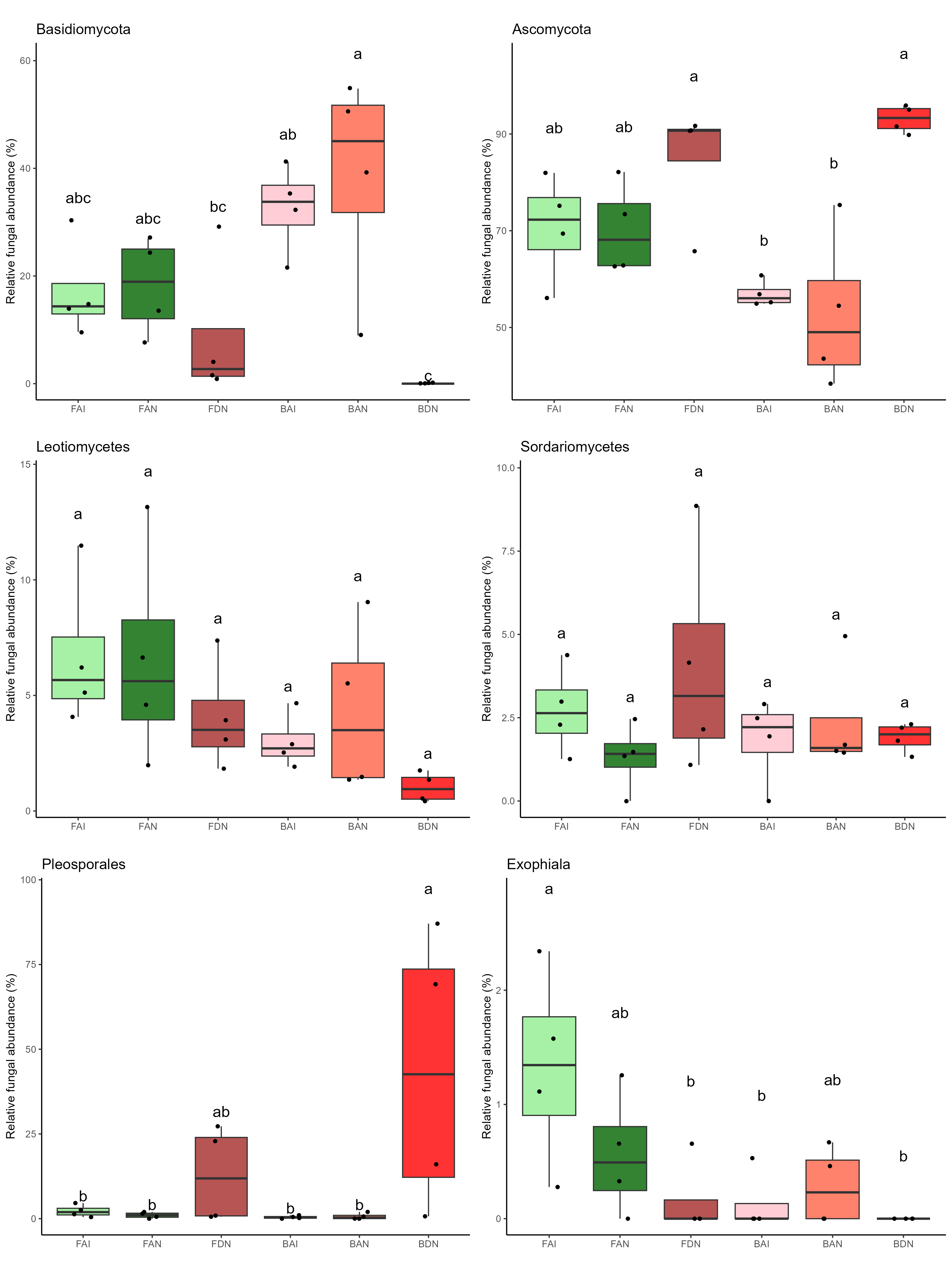

Supplement: Supplementary file 7 — Supplementary Material 7. [file 40793_2026_904_MOESM7_ESM.png]
