# Supplementary material for: Microbiome legacy influences necrosis formation in Diplodia sapinea-infected Scots pine shoots
Source: Environ Microbiome. 2026 May 9;21:64. doi: 10.1186/s40793-026-00904-9 (PMC13162409; doi:10.1186/s40793-026-00904-9)
Supplement: Supplementary file 9 — Supplementary Material 9. [file 40793_2026_904_MOESM9_ESM.docx]

**Supplementary material**

**Figure S1.** Overview of the EPI2ME workflow using Kraken2 and Bracken that has been applied in the present study.

**Supplementary Figure S2.** Comparison of previously covered (BAG) and free (FREE) shoots of Scots pine saplings before the inoculation experiments by reflectance and morphological features. **a**: Pictograms of the differently treated shoots visually explaining the color code and the status of the examined shoots. Principal component analysis (**b**), and boxplots (**c**) show values of the 64 default parameters of the Phenospex planteye multispectral 3D scanner derived from previously covered (BAG) and free (FREE) shoots of Scots pine saplings before the inoculation experiments. T-tests with Benjamini-Hochberg correction showed no significant difference between the groups, hence letters above the boxes are not shown. Statistical summaries are provided in Table S1.

**Supplementary Figure S3.** Box plots showing the necrotic area and lesion perimeter in shoots of *Pinus sylvestris* previously covered with mesh bags (BAG) or left uncovered (FREE) and inoculated with *Diplodia sapinea*. **a**: Necrotic area measured in the samples used for microbiome analysis. **b**: Lesion perimeter measured in the samples used for microbiome analysis. **c**: Necrotic area measured when all inoculated samples were included. **d**: Lesion perimeter measured when all inoculated samples were included. P values shown in the plots refer to t-tests with Benjamini–Hochberg correction (see Table S1).

**Supplementary Figure S4.** Rarefaction curves representing the 4131 reads of each of the 24 samples used in this study, showing the early reach of a plateau. Different colors represent the certain samples (Pin01–Pin24, see Suppl. Table S1).

**Supplementary Figure S5.** Non-metric multidimensional scaling (NMDS) ordination plots showing the differences in community composition of various treatment groups and lifestyles of fungi in the Scots pine shoots. Different colors represent the sample groups: Intact shoot parts below the agar-inoculated covered (bag-agar-intact, BAI) and free shoots (free-agar-intact, FAI), agar-inoculated shoot parts of covered (bag-agar-necro, BAN) and free shoots (free-agar-necro, FAN), and parts inoculated with *D. sapinea* in the case of covered (bag-*Diplodia*-necro, BDN) and free shoots (free-*Diplodia*-necro, FDN). Statistical summaries are provided in Table S1.

**Figure S6.** Heat trees showing phylogenetic composition of fungal communities in differently treated and inoculated Scots pine shoots. The different treatment groups of the shoots previously covered at the site are shown in green frame, those of shoots previously covered with mesh bags are shown within dark red frame. Each node represents a taxon from kingdom to genera, and only the taxa representing more than 0.05% of the total fungal reads were visualized in the heat tree. Tree topology is the same for every sample, whereas color of the nodes goes from purple (100% relative abundance), through red, orange and green to yellow (0% relative abundance) according to abundance of the taxa in each sample. Small numbers on the edges give abundance of the taxa in read numbers, which follow the given node.

**Supplementary Figure S7.** Boxplots showing the distribution of relative abundance values for each fungal taxa across the six treatment groups: Intact shoot parts (FAI), agar-inoculated areas (FAN) and *Diplodia sapinea*-inoculated necrotic parts (FDN) of previously free Scots pine shoots, and Intact shoot parts (BAI), agar-inoculated areas (BAN) and *D. sapinea*-inoculated necrotic parts (BDN) of previously covered Scots pine shoots. Letters above the boxes indicate statistically significant differences among groups as determined by one-way ANOVA followed by Tukey’s HSD post hoc test. Groups not sharing a letter are significantly different (p < 0.05). ANOVA summary statistics and Tukey pairwise comparisons are provided in Table S1.

**Supplementary Table S1**

Supplementary Material comprising the taxon table containing subsampled reads per sample used for analyses, statistics, and raw data on the different sheets.
